# Bone strain index in the prediction of vertebral fragility refracture

**DOI:** 10.1186/s41747-020-00151-8

**Published:** 2020-04-09

**Authors:** Fabio Massimo Ulivieri, Luca Petruccio Piodi, Luca Rinaudo, Paolo Scanagatta, Bruno Mario Cesana

**Affiliations:** 1 Fondazione IRCCS Ca’ Granda Ospedale Maggiore Policlinico, UO Medicina Nucleare, Via Francesco Sforza, 35, 20122 Milano, Italy; 2 Formerly: Fondazione IRCCS Ca’ Granda Ospedale Maggiore Policlinico, UO Gastroenterologia ed Endoscopia Digestiva, Via Francesco Sforza, 35, 20122 Milano, Italy; 3 TECHNOLOGIC Srl, Lungo Dora Voghera, 34/36A, 10153 Torino, Italy; 4 Scuola di Specializzazione in Medicina Fisica e Riabilitativa, Università degli Studi di Milano, Via Festa del Perdono, 7, 20122 Milano, Italy; 5 Unità di Statistica Medica, Biometria e, Bioinformatica “Giulio A. Maccacaro”, Dipartimento di Scienze Cliniche e Salute della Comunità, Università degli Studi di Milano, Via Vanzetti 5, 20100 Milano, Italy

**Keywords:** Bone density, Bone fractures, Absorptiometry (dual-energy x-ray), Finite element analysis, Osteoporosis

## Abstract

Dual-energy x-ray absorptiometry (DXA) can provide quantitative (bone mineral density, BMD) and qualitative (trabecular bone score, TBS) indexes of bone status, able to predict fragility fractures in most osteoporotic patients. A new qualitative index of bone strength, based on finite element analysis and named bone strain index (BSI), has been recently developed from lumbar DXA scan. We present the preliminary results about the BSI ability to predict a refracture in patients with fragility fractures. A total of 143 consecutive fractured patients with primary osteoporosis (121 females) performed a spine x-ray examination for the calculation of spine deformity index (SDI) and a DXA densitometry for BMD, TBS, and BSI at basal time and in the follow-up. A refracture was considered as a one-unit increase in SDI. For each unit increase of the investigated indexes, the hazard ratio of refracture, 95% confidence interval, *p* value, and proportionality test *p* value were for BSI 1.201, 0.982−1.468, 0.074, and 0.218; for lumbar BMD 0.231, 0.028−1.877, 0.170, and 0.305; and for TBS 0.034, 0.001−2.579, 0.126, and 0.518, respectively. BSI was the index predictive of refracture nearest to statistical significance. If confirmed, it may be used for a better risk assessment of osteoporotic patients.

## Key points

- Bone strain index (BSI) is a new promising index of bone strength derived from dual-energy x-ray absorptiometry (DXA).
- BSI shows the potential to be used in the setting of predicting fragility refracture.
- BSI in conjunction with other DXA bone quantity and quality parameters could ameliorate the fragility fracture risk assessment.

## Background

Dual-energy x-ray absorptiometry (DXA) is the standard method to assess bone status in osteoporosis as defined by the World Health Organization [1]. It provides quantitative and qualitative data as bone mineral density (BMD) and trabecular bone score (TBS), respectively. Although fracture risk is closely related to BMD, many of the fractured patients do not present a relevant reduction in BMD [2]. Moreover, patients affected by secondary osteoporosis, *e.g*., due to diabetes, suffer from fragility fractures with normal or slightly reduced BMD, but may present low TBS [3]. TBS is a grey level textural-architectural index derived from lumbar spine DXA able to predict fragility fractures also independently from BMD in retrospective and longitudinal studies [4]. However, TBS does not provide appropriate indications about bone strength and fatigue that characterise the resistance of a structure to loads over time [5].

A new bone quality index has been recently developed, currently being validated, defined as *bone strain index* (BSI) [6, 7], an index of bone strength, which appears to be promising in the characterisation of osteoporotic patients prone to fracture [8, 9]. It is a representation of the internal vertebral strain calculated with finite element analysis. This mathematical approach is used in engineering to solve problems related to complex geometry domains, such as the shape and the internal structure of bone tissue, and is based on the theory that by dividing an object into smaller and simpler elements it is possible to easily find the solution of a system of differential algebraic equations relating the internal stress and strain of the bone. Looking forward to assess the validity of BSI in characterising osteoporotic patients as a diagnostic, therapeutic and fractures predictive tool, we would like to anticipate preliminary data about the methodology of BSI determination and its ability to predict a refracture in a sample of patients with fragility fractures.

## Methods

Among the osteoporotic outpatients followed by the Bone Metabolic Unit at Fondazione IRCCS Ca’ Granda Ospedale Maggiore Policlinico of Milan, Italy, 143 consecutive patients fulfilling the inclusion/exclusion criteria were enrolled in this study. Inclusion criterion was the presence of a fragility fracture within 1 year before. Exclusion criteria were bone diseases or pharmacological treatments known to interfere with bone metabolism, traumatic and pathological fractures. Treatment for osteoporosis was not an exclusion criterion. All patients underwent yearly spine x-ray examination and DXA bone densitometry every 2 years after baseline. All patients signed a written informed consent and local Ethical Committee approval was obtained (Comitato Etico Milano Area 2. Protocol N 2.0 BQ. 265_2017, 13th June 2017).

The spine x-ray evaluation was performed for the assessment of the spine deformity index (SDI) [10]. We considered the worsening of the SDI by one unit as the expression of a refracture.

Lumbar spine DXA bone densitometry (Hologic Discovery A system, Hologic Inc., Marlborough, MA, USA; software version 13.3.0.1) was performed in order to obtain bone quantity and quality indexes: lumbar spine BMD (g/cm^2^), TBS and BSI.

For calculating BSI we should consider that an object that is constrained in the direction of the force will show a deformation. The extent of the deformation depends upon many factors like the magnitude and direction of the force as well as the material properties and the geometry of the object.

In this context, the calculation of BSI is obtained dividing each vertebra of the DXA lumbar scan into small triangular elements, with the load applied to upper surface and the constraints to the lower one. The solution of this system provides the deformation status of the vertebra with the related strain and stresses. In a previous study, the *in vitro* BSI least significant change appeared to be about three times that of BMD in all scan modalities and fat thicknesses interpositions [11].

**Fig. 1:**
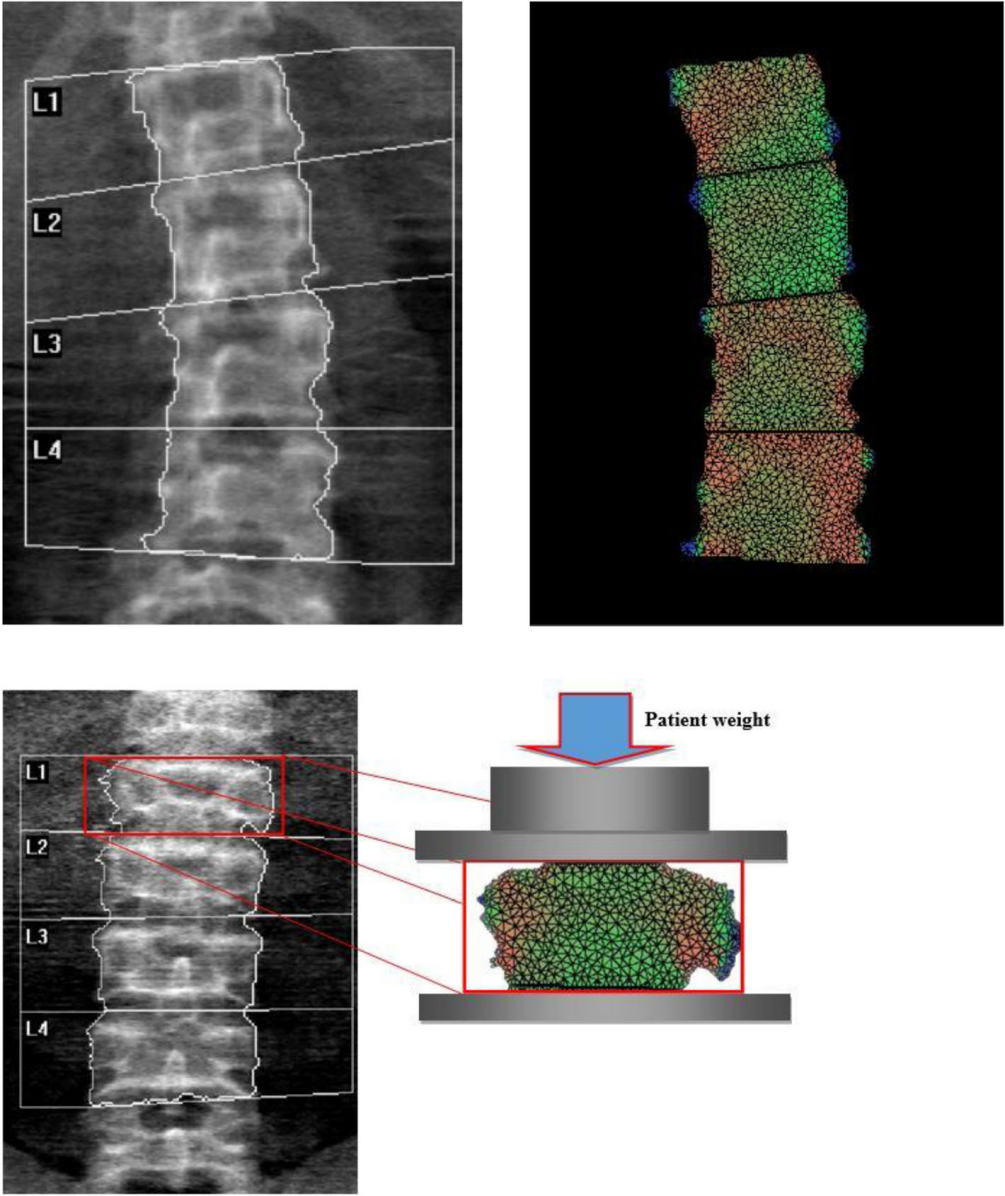
Example of a lumbar dual-energy x-ray absorptiometry (DXA) scan in the absence of fracture (upper left) and its bone strain index distribution (upper right). Example of a spine DXA scan (bottom left) and of bone strain index distribution for a fractured lumbar vertebra (bottom right) after distribution

The load applied to the vertebra is calculated specifically for each patient according to Han equations [12] and depends on patient’s weight and height, whereas the mechanical properties are defined in a stiffness matrix assigning elastic modulus depending on local BMD [7]. The BSI represents the level of the strain inside the vertebra, with the assumption that a higher strain level (*i.e*., a high BSI) indicates a greater risk condition. Figure 1 (upper left and right) shows an example of bone strain distribution within a lumbar DXA scan and the related BSI calculation. The image resulting from BSI algorithms shows the lumbar vertebrae of the patients divided in small triangular elements. The size of each element is regulated automatically by the Delaunay triangulation algorithm applied to the contour of the object, whereas the colour is proportional to the level of the strain calculated. The colour map follows a ramp from blue (low strain) to green (intermediate strain), yellow, and red (high strain) indicating an increase of the risk factor proportionated to the increase of the strain. In this way, it is simple to detect areas with high strain peaks that probably are more prone to fracture. The colour (strain level) of each triangle depends, in addition to the local features (*e.g.*, local BMD), on the BMD distribution around the element, the geometry of the object and the load applied on it.

BSI allows to consider all these features opportunely weighted, providing a way to analyse the data. Figure 1 bottom shows an example of bone strain distribution within a fractured lumbar spine vertebra.

### Statistical analysis

Mean, standard deviation (SD), median, and range have been calculated for quantitative variables; absolute and per cent frequencies have been calculated for qualitative variables. The two subgroups (refractured and not refractured) have been compared by means of the Student *t* test or Chi-square test in the case of quantitative or qualitative variables, respectively. A *p* value lower than 0.05 (two tailed) has been considered as statistically significant. The cumulative probability of not having a refracture has been estimated by the Kaplan-Meier product-limit method. The ability of the bone variables to predict refractures has been assessed by means of Cox proportionality hazard regression (SAS 9.2 version) and its proportionality assumption has been formally assessed by means of the proportionality test.

We note that with a sample size of 143 patients and 61 events, leading to an approximately conservative event rate of 0.30, a satisfactory power value (> 0.80) is obtained to demonstrate a hazard ratio at least of 1.35.

## Results

Of the 143 enrolled patients, 121 (84.6%) were female and 22 (15.4%) were male. Women’s mean age was 67.9 years (SD 10.9), significantly different (*p* = 0.043) from men’s age (60.0 years, SD15.5).

Among the 143 patients who had a fragility fracture at baseline, 61 had a refracture during the follow-up (mean 1154 days, median 864, range 320−3214). The SDI mean of the 143 patients was 4.31 (SD 4.32), the median 3 and the range 1−24.

**Fig. 2:**
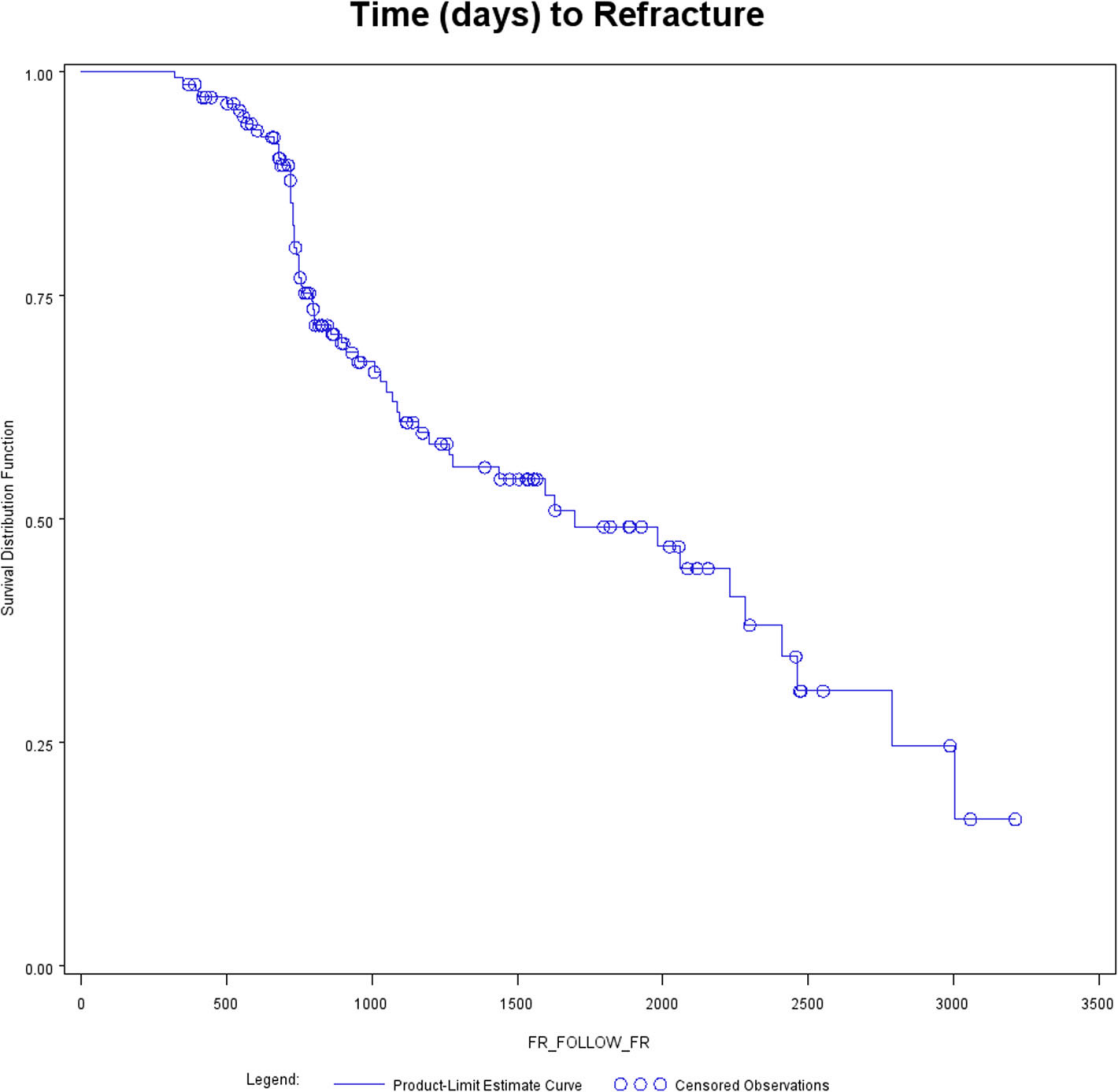
Kaplan-Meier estimate of the probability of not having a refracture

Table 1 reports the DXA results of the 143 patients divided by the refracture criterion as previously described. Only the TBS appeared significantly lower in the refractured patients. Figure 2 shows the Kaplan-Meier estimate of the probability of no refracture expressed as days from baseline to refracture. In particular, the probability of not having a refracture was 0.986 (95%CI 0.966−1.000) at 1 year of follow-up, 0.820 (0.752−0.888) at 2 years, 0.608 (0.514−0.702) at 3 years, and 0.545 (0.445−0.645) at 4 years. Then, the estimates have to be considered not reliable, being the at-risk remaining patients less than the 20% of the starting sample.

**Table 1:** Dual-energy x-ray absorptiometry in 143 patients with a fracture at baseline: bone strain index (BSI), lumbar bone mineral density (BMD) and trabecular bone score (TBS)

	Not refractured (*n* = 82)			Refractured (*n* = 61)			*p* value
	Mean ± SD	Median	Range	Mean ± SD	Median	Range	
BSI	4.978 ± 1.323	4.863	2.592–9.438	5.266 ± 1.389	5.077	2.463–9.130	0.2307
BMD (g/cm^2^)	0.773 ± 0.151	0.755	0.401–1.486	0.741 ± 0.141	0.722	0.450–1.160	0.2297
BMD males	0.874 ± 0.222	0.835	0.653–1.486	0.892 ± 0.165	0.847	0.664–1.160	0.7583
BMD females	0.749 ± 0.119	0.746	0.401–1.060	0.720 ± 0.126	0.701	0.450–1.017	0.1788
TBS	1.159 ± 0.097	1.176	0.982–1.365	1.053 ± 0.119	1.045	0.805–1.268	0.0016

*SD* Standard deviation

In addition, Table 2 reports the hazard ratio of refracture, 95% confidence intervals, *p* value and proportionality test *p* value for each unit increase in the investigated indexes.

**Table 2:** Dual-energy x-ray absorptiometry in 143 patients with a fracture at baseline. Statistical results for bone strain index (BSI), lumbar bone mineral density (BMD) and trabecular bone score (TBS): hazard ratio of refracture, 95% confidence interval, *p* value, and proportionality test *p* value for each unit increase in the investigated indexes.

	Hazard ratio	95% confidence interval	*p* value	Proportionality *p* value
BSI	1.201	0.982–1.468	0.0739	0.218
BMD	0.231	0.028–1.877	0.1703	0.305
TBS	0.034	0.001–2.579	0.1257	0.518

## Discussion

In osteoporotic patients with fragility fractures there is a high risk of a refracture following an initial fracture and this risk is exponentially related with the number of previous fractures and with the severity of them [13]. The therapy can reduce the initial risk by about 50%, but does not reset it. In a retrospective cohort study, the treated arm began to diverge from the placebo one after at least 6 months and the maximum reduction in fracture risk was reached after at least 1 or 2 years of treatment [14]. Furthermore, patients commonly begin drug treatment at the end of the diagnostic procedure, which often takes a long time after the first fracture. For these reasons, prediction of a refracture is mandatory for a prompt management to avoid this event, which is dramatic for patients’ quality of life. In addition, since the patients’ adherence to drug treatment is very low (about 30% at 1 year [15]), the knowledge of the risk of refracture could improve the adherence.

In this study, of the three considered DXA indexes, the BSI resulted to be the nearest to the statistical significance to predict a refracture, with greater values associated to higher refracture risk.

The TBS’ lack of significance in refracture prediction is very likely due to the number of missing data and to the moderate number of patients of our sample, since its capability to predict fracture has been demonstrated in literature [16]. However, in our study, TBS turned out to be significantly different between the two subgroups at baseline, being in the refractured group lower than in the not refractured one. Also, BMD did not show significance in refracture prediction, but, as aforementioned, many of the fractured patients do not present relevant variations in BMD. Fracture is related with the material properties of bone, well described by BMD and TBS, being these variables directly involved in material density and structure. Indeed, material properties are among the main features that define the risk of fracture of an object, but even geometry and load should be considered. BSI seems to be more suitable for analysing irregular, complex structures, among which fractured bone may be included. Thus, if confirmed in larger studies, also BSI may be used in association with the other DXA-derived bone quantity and quality variables for a better risk assessment of osteoporotic patients.

## Data Availability

The datasets used and analysed during the current study are available from the corresponding author on reasonable request.

## Abbreviations

BSI: Bone strain index
BMD: Bone mineral density
DXA: Dual-energy x-ray absorptiometry
SDI: Spine deformity index
TBS: Trabecular bone score
